# Supplementary material for: RiboTRAP-seq identifies spatially distinct functions for the anterior and posterior intestine in immune and metabolic regulation in C. elegans
Source: bioRxiv. 2025 Oct 4:2025.10.03.680215. Preprint. [Version 1] doi: 10.1101/2025.10.03.680215 (PMC12621888; doi:10.1101/2025.10.03.680215)
Supplement: Supplement 1 [file NIHPP2025.10.03.680215v1-supplement-1.pdf]

bioRxiv preprint doi: https://doi.org/10.1101/2025.10.03.680215; this version posted October 4, 2025. The copyright holder for this preprint (which was not certified by peer review) is the author/funder, who has granted bioRxiv a license to display the preprint in perpetuity. It is made available under aCC-BY 4.0 International license.

S1 Fig

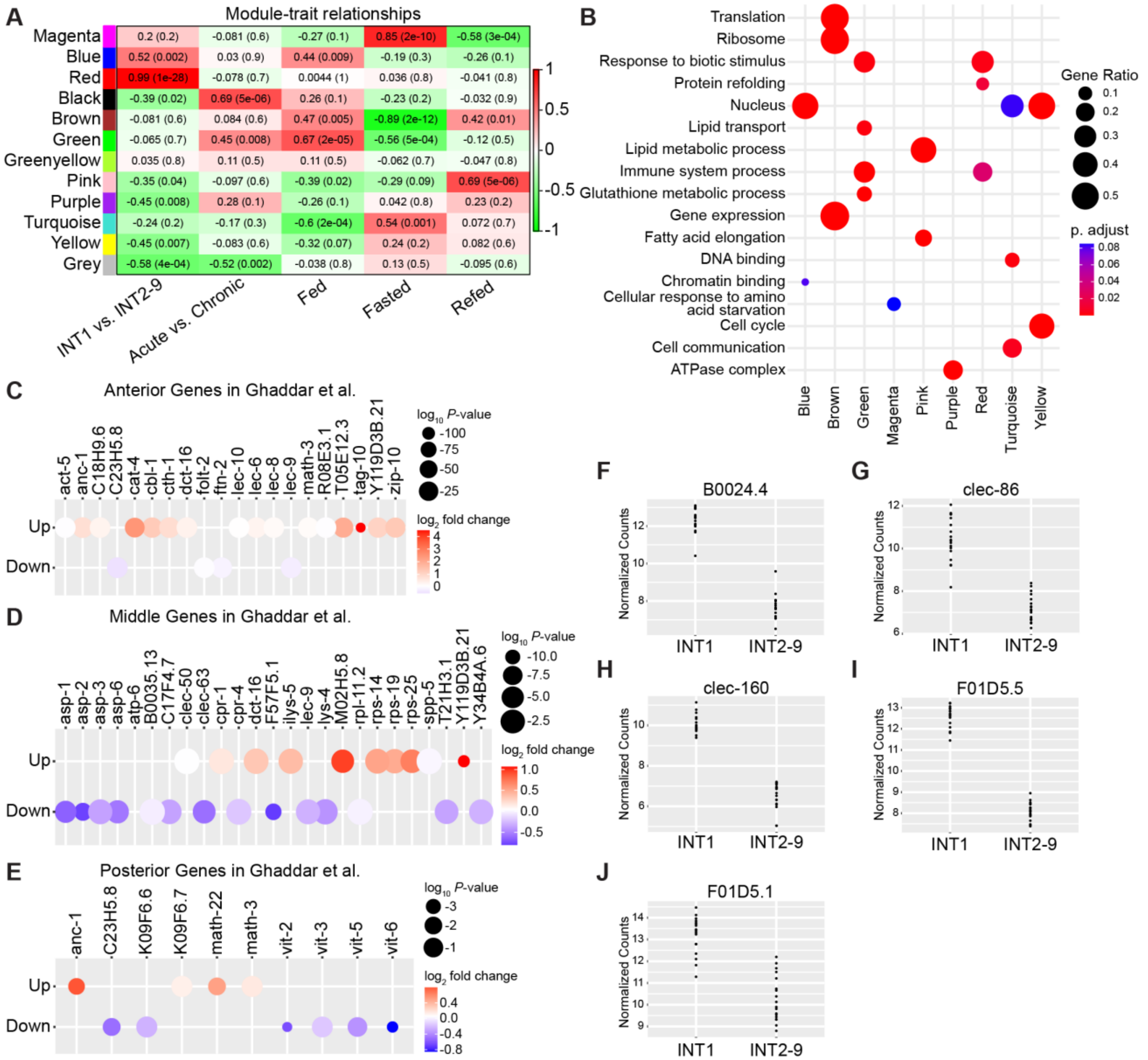

S1 Fig. The genes differentially expressed in INT1 cells.

(A) Module-trait relationships for selected conditions in WGCNA. Rows indicate the WGCNA module and the columns comparative traits. The color indicates the strength of the pearsons correlation and bracketed number the significance. The color red represents a positive correlation; the color green represents a negative correlation. (B) GO enrichment of selected modules in the WGCNA analysis. Size indicates the strength of the significance and color the significance. (C-E) Plots showing the fold change of the intestinal anterior, middle and posterior genes from Ghaddar et al. in our INT1 vs. INT2-9 Fed datasets. (F-J) Expression profiles of *B0024.4, clec-86, clec-160, F01D5.5* and *F01D5.1* under all conditions in INT1 and INT2-9 cells.

bioRxiv preprint doi: https://doi.org/10.1101/2025.10.03.680215; this version posted October 4, 2025. The copyright holder for this preprint (which was not certified by peer review) is the author/funder, who has granted bioRxiv a license to display the preprint in perpetuity. It is made available under aCC-BY 4.0 International license.

S2 Fig

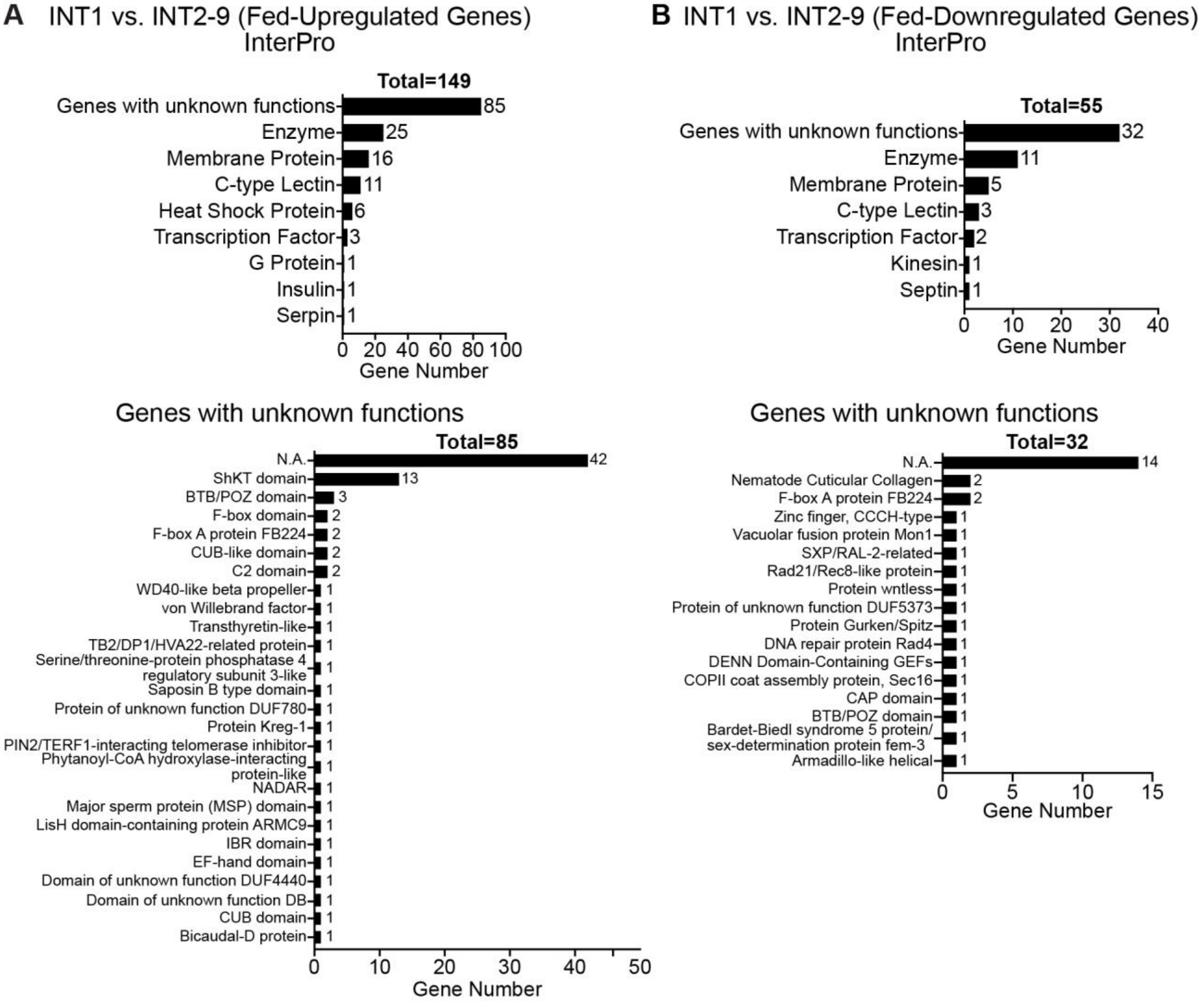

S2 Fig. InterPro annotations of differentially expressed genes for INT1 Fed versus INT2-9 Fed.

(A, B) Bar charts of the upregulated or downregulated genes categorized by the protein domains predicted by InterPro for INT1 fed versus INT2-9 fed.

bioRxiv preprint doi: https://doi.org/10.1101/2025.10.03.680215; this version posted October 4, 2025. The copyright holder for this preprint (which was not certified by peer review) is the author/funder, who has granted bioRxiv a license to display the preprint in perpetuity. It is made available under aCC-BY 4.0 International license.

S3 Fig

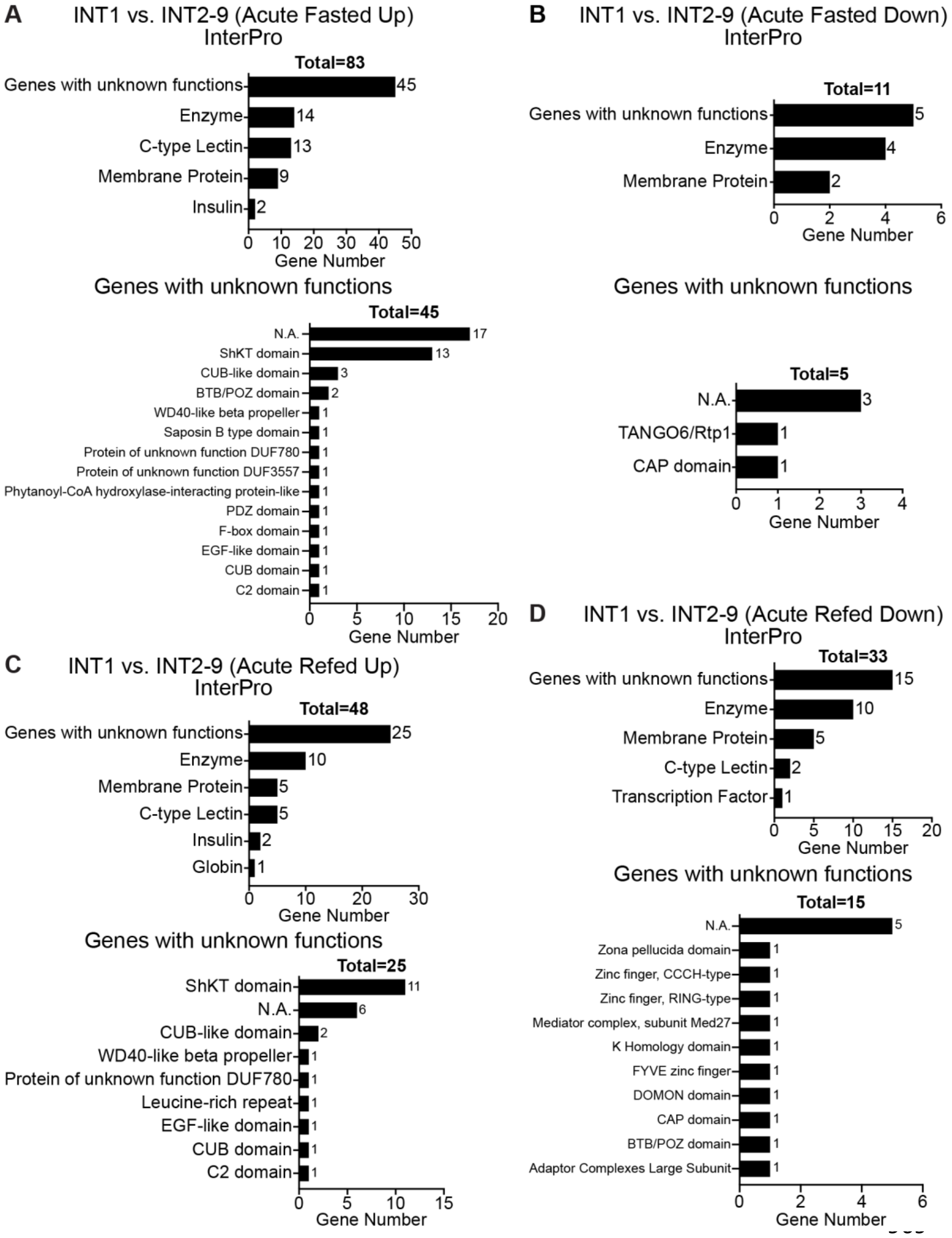

S3 Fig. InterPro annotations of differentially expressed genes for INT1 versus INT2-9 under acute conditions.

bioRxiv preprint doi: https://doi.org/10.1101/2025.10.03.680215; this version posted October 4, 2025. The copyright holder for this preprint (which was not certified by peer review) is the author/funder, who has granted bioRxiv a license to display the preprint in perpetuity. It is made available under aCC-BY 4.0 International license.

(A, B) Bar charts of the upregulated or downregulated genes categorized by the protein domains predicted by InterPro for INT1 30 min fasted versus INT2-9 30 min fasted. (C, D) Bar charts of the upregulated or downregulated genes categorized by the protein domains predicted by InterPro for INT1 30 min refed versus INT2-9 30 min refed.

bioRxiv preprint doi: https://doi.org/10.1101/2025.10.03.680215; this version posted October 4, 2025. The copyright holder for this preprint (which was not certified by peer review) is the author/funder, who has granted bioRxiv a license to display the preprint in perpetuity. It is made available under aCC-BY 4.0 International license.

S4 Fig

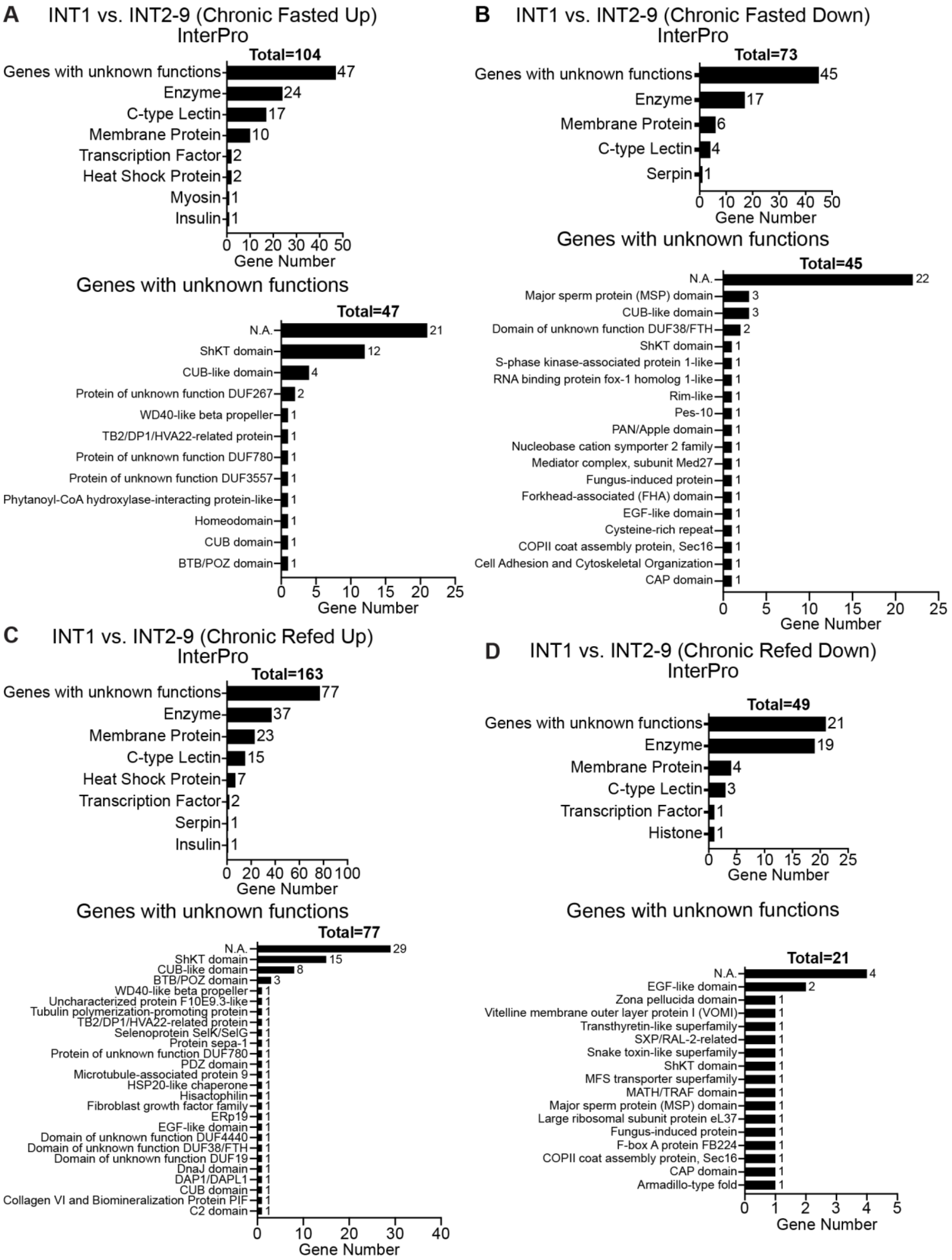

bioRxiv preprint doi: https://doi.org/10.1101/2025.10.03.680215; this version posted October 4, 2025. The copyright holder for this preprint (which was not certified by peer review) is the author/funder, who has granted bioRxiv a license to display the preprint in perpetuity. It is made available under aCC-BY 4.0 International license.

S4 Fig. InterPro annotations of differentially expressed genes for INT1 versus INT2-9 under chronic conditions.

(A, B) Bar charts of the upregulated or downregulated genes categorized by the protein domains predicted by InterPro for INT1 180 min fasted versus INT2-9 180 min fasted. (C, D) Bar charts of the upregulated or downregulated genes categorized by the protein domains predicted by InterPro for INT1 90 min refed versus INT2-9 90 min refed.

bioRxiv preprint doi: https://doi.org/10.1101/2025.10.03.680215; this version posted October 4, 2025. The copyright holder for this preprint (which was not certified by peer review) is the author/funder, who has granted bioRxiv a license to display the preprint in perpetuity. It is made available under aCC-BY 4.0 International license.

S5 Fig

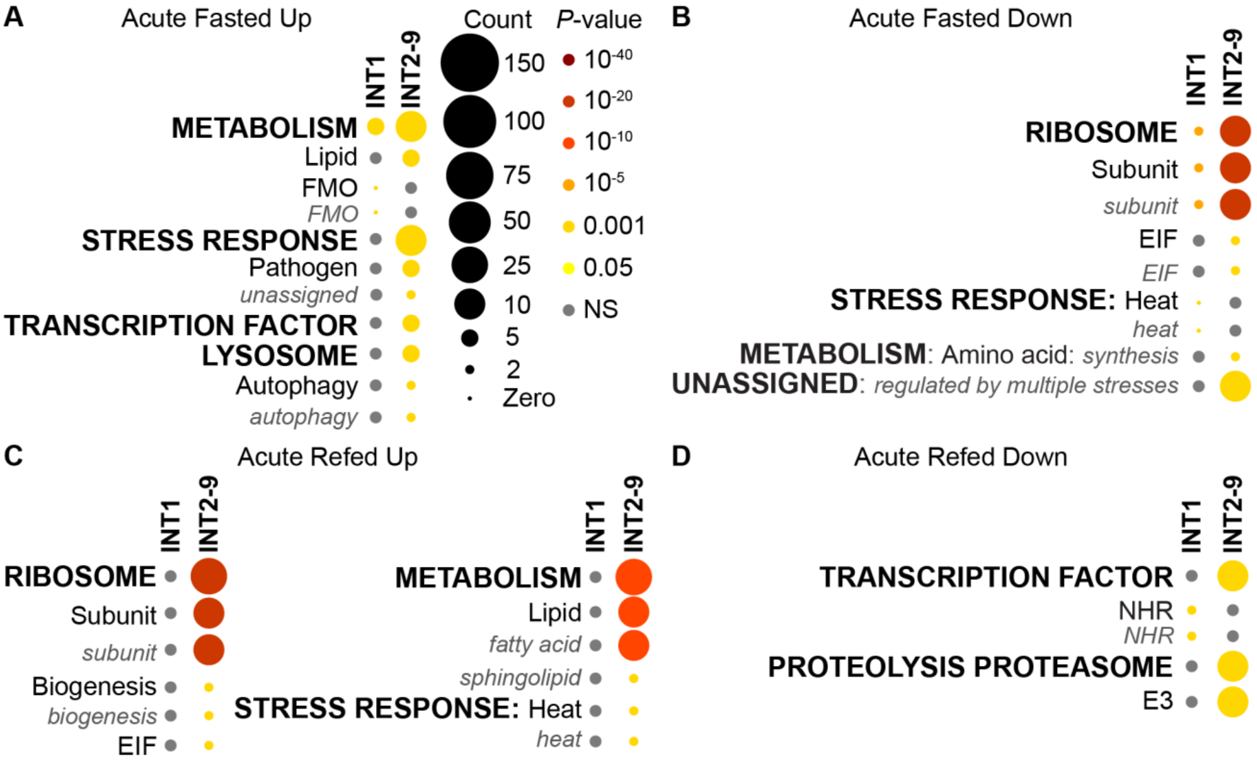

S5 Fig. WormCat annotations of differentially expressed genes under acute fasting and refeeding conditions for INT1 and INT2-9 cells.

(A, B) WormCat visualization of categories enriched in differentially expressed genes in INT1 cells and INT2-9 cells under acute fasting condition. (C, D) WormCat visualization of categories enriched in differentially expressed genes in INT1 cells and INT2-9 cells under acute refeeding condition. Categories 1 are all bold uppercase; Categories 2 are capitalized; Categories 3 are gray italics. The size of the bubbles indicates the gene counts in the category and the color of the bubble represents the adjusted p-value.

bioRxiv preprint doi: https://doi.org/10.1101/2025.10.03.680215; this version posted October 4, 2025. The copyright holder for this preprint (which was not certified by peer review) is the author/funder, who has granted bioRxiv a license to display the preprint in perpetuity. It is made available under aCC-BY 4.0 International license.

S6 Fig

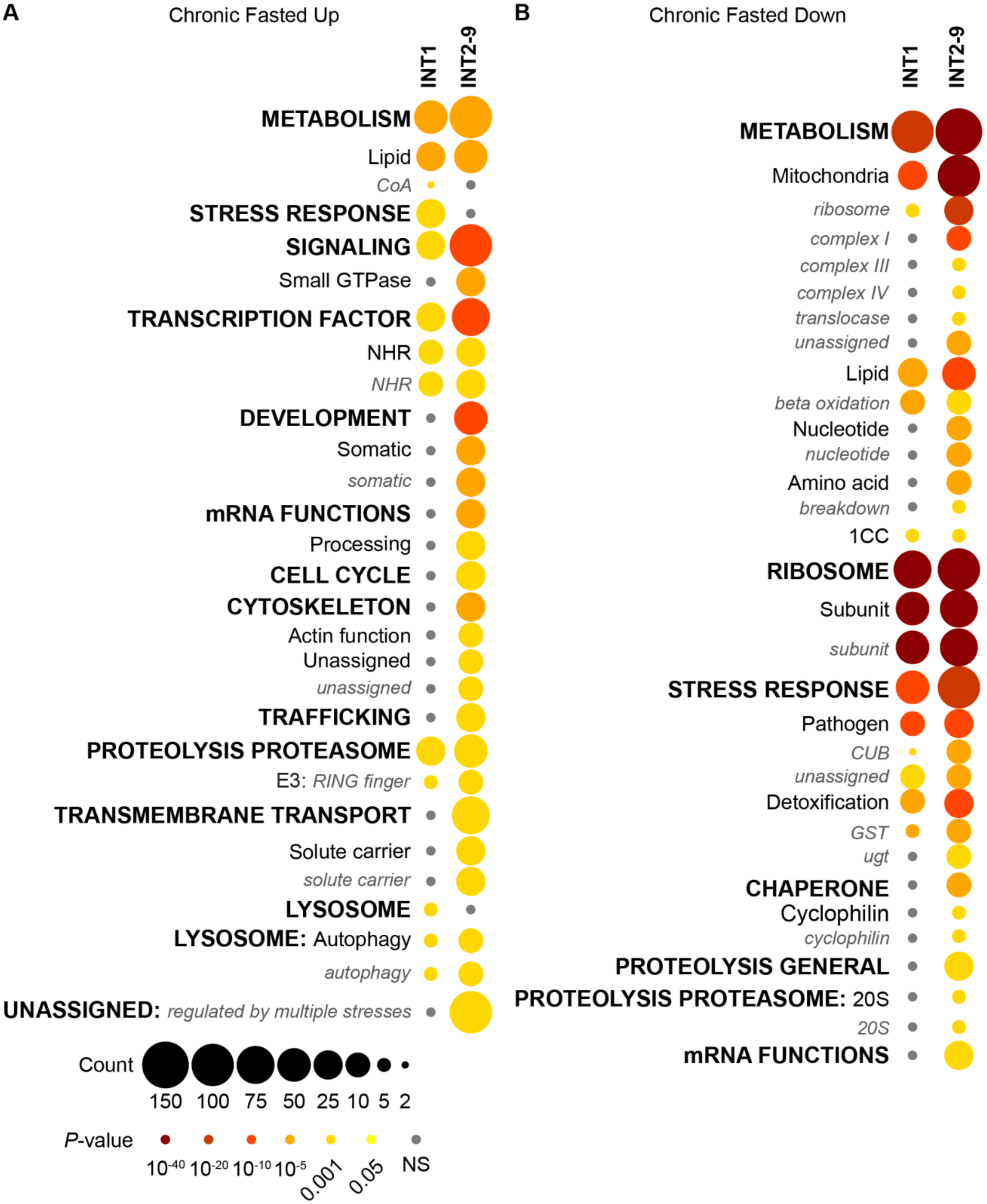

1038 **S6 Fig. WormCat annotations of differentially expressed genes under chronic fasting**
1039 **condition for INT1 and INT2-9 cells.**

bioRxiv preprint doi: https://doi.org/10.1101/2025.10.03.680215; this version posted October 4, 2025. The copyright holder for this preprint (which was not certified by peer review) is the author/funder, who has granted bioRxiv a license to display the preprint in perpetuity. It is made available under aCC-BY 4.0 International license.

(A, B) WormCat visualization of categories enriched in differentially expressed genes in INT1 cells and INT2-9 cells under chronic fasting condition. Categories 1 are all bold uppercase; Categories 2 are capitalized; Categories 3 are gray italics. The size of the bubbles indicates the gene counts in the category and the color of the bubble represents the adjusted p-value.

bioRxiv preprint doi: https://doi.org/10.1101/2025.10.03.680215; this version posted October 4, 2025. The copyright holder for this preprint (which was not certified by peer review) is the author/funder, who has granted bioRxiv a license to display the preprint in perpetuity. It is made available under aCC-BY 4.0 International license.

S7 Fig

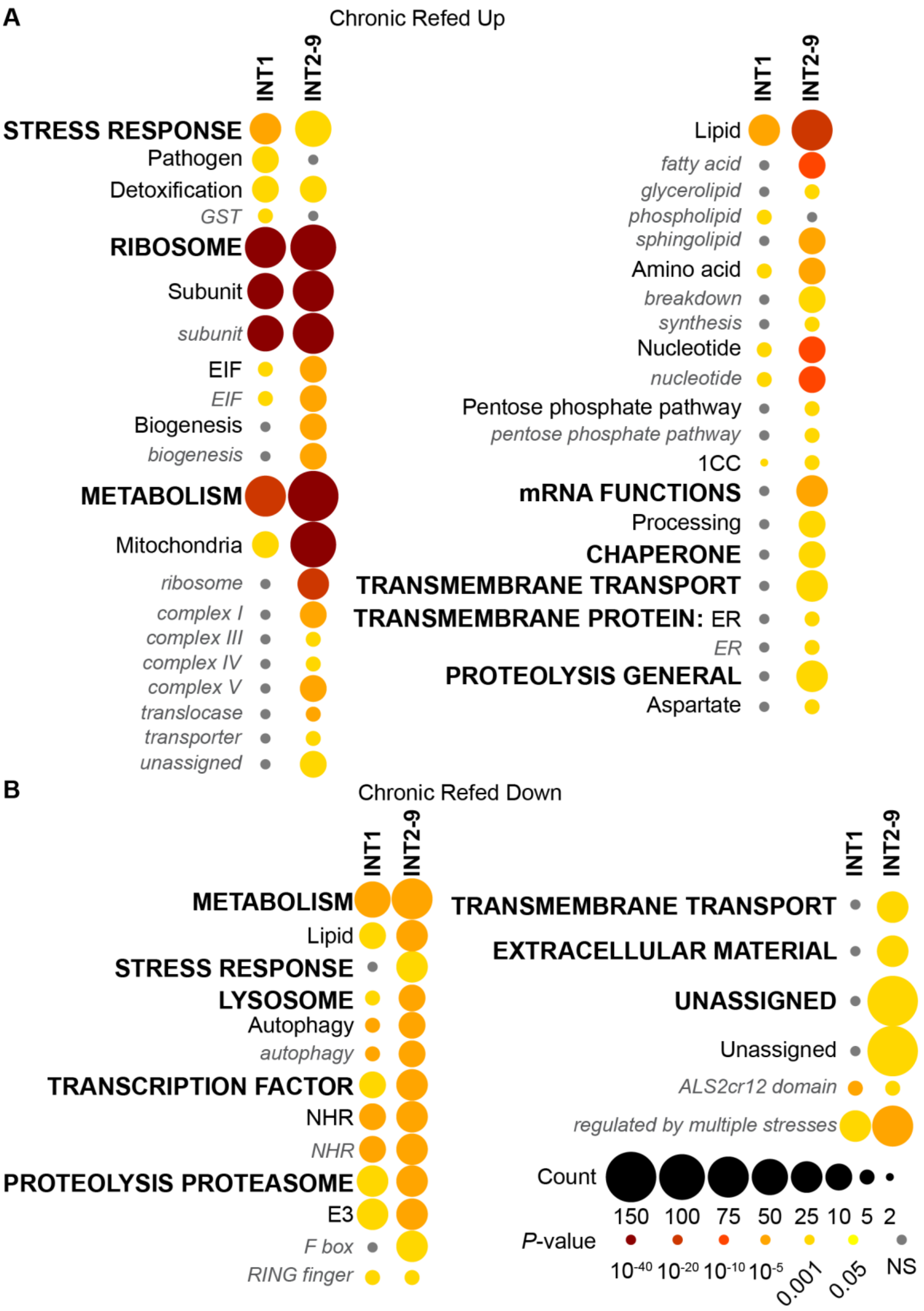

bioRxiv preprint doi: https://doi.org/10.1101/2025.10.03.680215; this version posted October 4, 2025. The copyright holder for this preprint (which was not certified by peer review) is the author/funder, who has granted bioRxiv a license to display the preprint in perpetuity. It is made available under aCC-BY 4.0 International license.

S7 Fig. WormCat annotations of differentially expressed genes under chronic refeeding condition for INT1 and INT2-9 cells.

(A, B) WormCat visualization of categories enriched in differentially expressed genes in INT1 cells and INT2-9 cells under chronic refeeding condition. Categories 1 are all bold uppercase; Categories 2 are capitalized; Categories 3 are gray italics. The size of the bubbles indicates the gene counts in the category and the color of the bubble represents the adjusted p-value.

bioRxiv preprint doi: https://doi.org/10.1101/2025.10.03.680215; this version posted October 4, 2025. The copyright holder for this preprint (which was not certified by peer review) is the author/funder, who has granted bioRxiv a license to display the preprint in perpetuity. It is made available under aCC-BY 4.0 International license.

S8 Fig

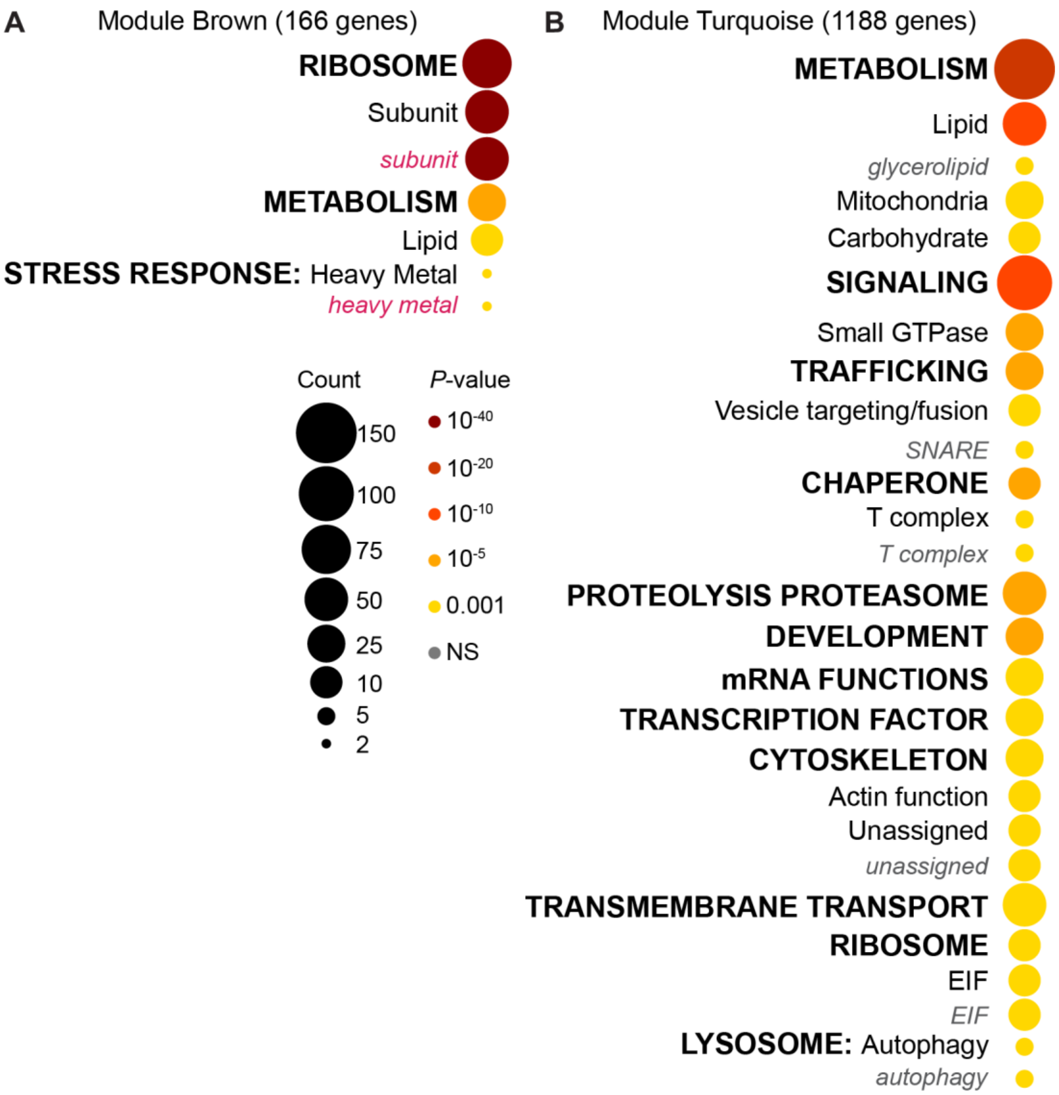

S8 Fig. WormCat annotations of undulating genes in INT1 and INT2-9 cells.

(A, B) WormCat visualization of categories enriched in genes of module brown and turquoise. Categories 1 are all bold uppercase; Categories 2 are capitalized; Categories 3 are gray italics. The size of the bubbles indicates the gene counts in the category and the color of the bubble represents the adjusted p-value.

bioRxiv preprint doi: https://doi.org/10.1101/2025.10.03.680215; this version posted October 4, 2025. The copyright holder for this preprint (which was not certified by peer review) is the author/funder, who has granted bioRxiv a license to display the preprint in perpetuity. It is made available under aCC-BY 4.0 International license.

S9 Fig

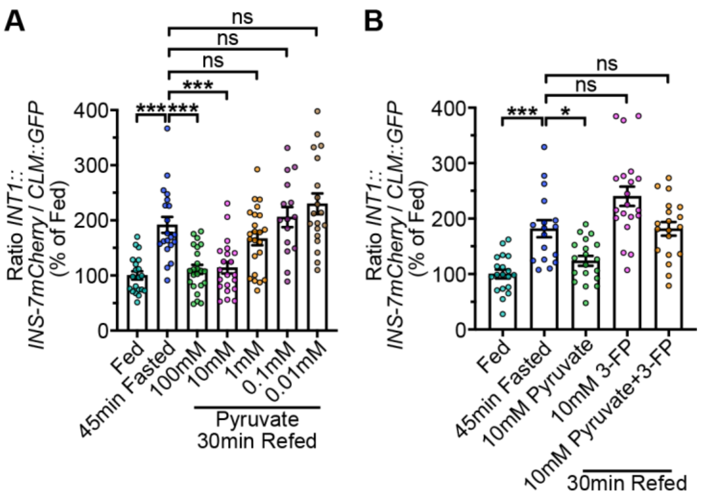

S9 Fig. The role of pyruvate in refeeding responses of INS-7 secretion from INT1 cells.

(A) INS-7 secretion dynamics during fasting and re-feeding with different concentrations of pyruvate (0.01 mM - 100 mM) were determined at the indicated time points. The intensity of INS-7mCherry fluorescence within a single coelomocyte was quantified and normalized to the area of CLM::GFP expression for each time point. Data are expressed as a percentage of the normalized INS-7mCherry fluorescence intensity of wild-type fed animals ± SEM (n=15-23). ***$p<0.001$, $^{ns}p>0.05$ by one-way ANOVA (Dunnett T3). (B) INS-7 secretion dynamics during fasting and re-feeding with 10 mM pyruvate, 10 mM 3-FP, and 10 mM pyruvate and 3-FP were determined at the indicated time points. The intensity of INS-7mCherry fluorescence within a single coelomocyte was quantified and normalized to the area of CLM::GFP expression for each time point. Data are expressed as a percentage of the normalized INS-7mCherry fluorescence intensity of wild-type fed animals ± SEM (n=17-20). *$p<0.05$, ***$p<0.001$, $^{ns}p>0.05$ by one-way ANOVA (Dunnett T3).

bioRxiv preprint doi: https://doi.org/10.1101/2025.10.03.680215; this version posted October 4, 2025. The copyright holder for this preprint (which was not certified by peer review) is the author/funder, who has granted bioRxiv a license to display the preprint in perpetuity. It is made available under aCC-BY 4.0 International license.

Supplementary Table 1: *C. elegans* strains used in this study.

Strains used in this study	Source	Identifier	Additional Information
N2; ssrIs1248[Pges-1ΔB::rpl-22-3xHA]; ssrIs1191[Pges-1ΔB::GFP]	This paper	SSR1578	Integrated. Backcrossed 4X.
N2; ssrIs1191[Pges-1ΔB::GFP]; ssrIs1210[Ppho-1::mCherry]	This paper	SSR1582	Integrated. Backcrossed 4X.
N2; ssrIs1251[Ppho-1::rpl-22-3xHA]; ssrIs1210[Ppho-1::mCherry]	This paper	SSR1605	Integrated. Backcrossed 4X.
sid-1(qt9); ssrIs1220[Pges-1ΔB::sid-1::GFP]; ssrIs1240[Pges-1ΔB::ins-7mCherry]; ssrIs615[Punc-122::GFP]	PMID: 37961386	SSR1634	
N2; ssrEx1317[Pclec-160::mNeonGreen]; ssrEx1031[Pmyo-2::mCherry]	This paper	SSR1701	
N2; ssrEx1319[Pclec-86::mNeonGreen]; ssrEx1031[Pmyo-2::mCherry]	This paper	SSR1702	
N2; ssrEx1318[PB0024.4::mNeonGreen]; ssrEx1031[Pmyo-2::mCherry]	This paper	SSR1703	
N2; ssrEx1323[PF01D5.5::mNeonGreen]; ssrEx1031[Pmyo-2::mCherry]	This paper	SSR1705	
N2; ssrEx1324[Pnpr-28::mNeonGreen]; ssrEx1031[Pmyo-2::mCherry]	This paper	SSR1706	
N2; ssrEx1325[PF01D5.1::mNeonGreen]; ssrEx1031[Pmyo-2::mCherry]	This paper	SSR1713	

Supplementary Table 2: Plasmids used in this study.

Plasmids used in this study	Source	Identifier
Pges-1ΔB::GFP	This paper	pSS1191
Ppho-1::mCherry	This paper	pSS1210
Pges-1ΔB::rpl-22-3xHA	This paper	pSS1248
Ppho-1::rpl-22-3xHA	This paper	pSS1251
Pclec-160::mNG::clec-160 3'UTR	This paper	pSS1317
PB0024.4::mNG::B0024.4 3'UTR	This paper	pSS1318
Pclec-86::mNG::clec-86 3'UTR	This paper	pSS1319
PF01D5.5::mNG::F01D5.5 3'UTR	This paper	pSS1323
Pnpr-28::mNG::npr-28 3'UTR	This paper	pSS1324
PF01D5.1::mNG::F01D5.1 3'UTR	This paper	pSS1325

Supplementary Table 3: Cloning primers used in this study.

Gibson Assembly	Primer	Source	Sequence
Pclec-160::mNG::clec-160 3'UTR	Forward primer for cloning the *Pclec-160* sequence	IDT	CATGCCTGCAAGGAAAAAATTGGAAGGGAAAGGAAAACT
	Reverse primer for cloning the *Pclec-160* sequence	IDT	TTGGAGACCATCTGAATAATTCTTGGGTATTAAAAAAAAGATTTTTGTACCTATTGG
	Forward primer for cloning the mNG sequence	IDT	AATTATTCAGATGGTCTCCAAGGGAGAGGAG
	Reverse primer for cloning the mNG sequence	IDT	GAATGACATCACTACTTGTAGAGCTCGTCCATTCC
	Forward primer for cloning the *clec-160 3'UTR* sequence	IDT	CTACAAGTAGTGATGTCATTCTCAATTTGAATTTAAACAGAATAAACTATTTTTAAAAGT
	Reverse primer for cloning the *clec-160 3'UTR* sequence	IDT	AATTCGAGCTCTCGCCTCAATTCTGTGATTCATAAAAAGTTCA
	Forward primer for cloning the pUC19 sequence	IDT	ATTGAGGCGAGAGCTCGAATTCACTGGCCG
	Reverse primer for cloning the pUC19 sequence	IDT	AATTTTTTCCTTGCAGGCATGCAAGCTTG
Pclec-86::mNG::clec-86 3'UTR	Forward primer for cloning the *Pclec-86* sequence	IDT	AGCTTGCATGCCTACCCGACCGGCCG
	Reverse primer for cloning the *Pclec-86* sequence	IDT	CCTTGGAGACCATTTTCAGTGTCCAGTAAAATAATAAATTTTATCTGCTTATCACAG
	Forward primer for cloning the mNG sequence	IDT	CTGGACACTGAAAATGGTCTCCAAGGGAGAGGAG
	Reverse primer for cloning the mNG sequence	IDT	TTCGAATCAAAAGCTACTTGTAGAGCTCGTCCATTCCC
	Forward primer for cloning the *clec-86 3'UTR* sequence	IDT	GCTCTACAAGTAGCTTTTGATTCGAAAGAAAATATTTATATATTATGAGCTAAATTGGAT
	Reverse primer for cloning the *clec-86 3'UTR* sequence	IDT	GTGAATTCGAGCTACCTGAGCATGCCGTTTGG

bioRxiv preprint doi: https://doi.org/10.1101/2025.10.03.680215; this version posted October 4, 2025. The copyright holder for this preprint (which was not certified by peer review) is the author/funder, who has granted bioRxiv a license to display the preprint in perpetuity. It is made available under aCC-BY 4.0 International license.

	Forward primer for cloning the pUC19 sequence	IDT	GGCATGCTCAGGTAGCTCGAATTCACTGGCCGTCGTTTTACA
	Reverse primer for cloning the pUC19 sequence	IDT	CGGCCGGTCGGGTAGGCATGCAAGCTTGGCGT
PB0024.4::mNG::B0024.4 3'UTR	Forward primer for cloning the *PB0024.4* sequence	IDT	CTTGCATGCCTGTTTAATTTTAAATGAGAAAATAATGATTTTTTGTCATTAAATTTAACT
	Reverse primer for cloning the *PB0024.4* sequence	IDT	CTTGGAGACCATTCTACTAGAAATAGAAAGTTCGTTTGGAATACCG
	Forward primer for cloning the mNG sequence	IDT	TTTCTAGTAGAATGGTCTCCAAGGGAGAGGAG
	Reverse primer for cloning the mNG sequence	IDT	ACAAATATCAACCTACTTGTAGAGCTCGTCCATTCCC
	Forward primer for cloning the *B0024.4 3'UTR* sequence	IDT	TCTACAAGTAGGTTGATATTTGTTTGTAATTACGGACTTATGGTGC
	Reverse primer for cloning the *B0024.4 3'UTR* sequence	IDT	AGTGAATTCGAGATTATTTGTGGCCATCCCCAACTATAAAAAT
	Forward primer for cloning the pUC19 sequence	IDT	CCACAAATAATCTCGAATTCACTGGCCGTCG
	Reverse primer for cloning the pUC19 sequence	IDT	TTAAAATTAAACAGGCATGCAAGCTTGGCGT
PF01D5.5::mNG::F01D5.5 3'UTR	Forward primer for cloning the *PF01D5.5* sequence	IDT	AGCTTGCATGCCCTGGAAATTGTTCGGGGATTTTTAATTTTTTTTTAAGC
	Reverse primer for cloning the *PF01D5.5* sequence	IDT	CCTTGGAGACCATCGCGAGCTTGAAGTTTTTCTTTGTT
	Forward primer for cloning the mNG sequence	IDT	TTCAAGCTCGCGATGGTCTCCAAGGGAGAGGAG
	Reverse primer for cloning the mNG sequence	IDT	ATTTTTGTAATTACTACTTGTAGAGCTCGTCCATTCC
	Forward primer for cloning the *F01D5.5 3'UTR* sequence	IDT	CTCTACAAGTAGTAATTACAAAAATATCTTTGGAATTGTAAATATTGTTATTTGAATAAA
	Reverse primer for cloning the *F01D5.5 3'UTR* sequence	IDT	ATTCGAGCTCGGTTGCCGTTCCCCTATCCTACCGTAC
	Forward primer for cloning the pUC19 sequence	IDT	AGGGGAACGGCAACCGAGCTCGAATTCACTGGCC
	Reverse primer for cloning the pUC19 sequence	IDT	GAACAATTTCCAGGGCATGCAAGCTTGGCGT
PF01D5.1::mNG::F01D5.1 3'UTR	Forward primer for cloning the *PF01D5.1* sequence	IDT	CAAGCTTGCATGCCTGGATAAAGAAAGCATGAATTTGAATTTTATATGTACACTATTCT
	Reverse primer for cloning the *PF01D5.1* sequence	IDT	TCCCTTGGAGACCATTTGTTTTCTGCAAAACTTAGTTTTTAGATAAAATGACTTTAAGAC
	Forward primer for cloning the mNG sequence	IDT	TTTGCAGAAAACAAATGGTCTCCAAGGGAGAGGAG
	Reverse primer for cloning the mNG sequence	IDT	AATAAAAAGATTTATCTACTTGTAGAGCTCGTCCATTCCC
	Forward primer for cloning the *F01D5.1 3'UTR* sequence	IDT	AGCTCTACAAGTAGATAAATCTTTTTATTTTAATTTTCATCGTAACTCGGTAGTAGAACA
	Reverse primer for cloning the *F01D5.1 3'UTR* sequence	IDT	CAGTGAATTCGAGCTCAGACACACTACACCTAACTCGTTCAG
	Forward primer for cloning the pUC19 sequence	IDT	GTGTAGTGTGTCTGAGCTCGAATTCACTGGCCG
	Reverse primer for cloning the pUC19 sequence	IDT	TGCTTTCTTTATCCAGGCATGCAAGCTTGGCGT
Pnpr-28::mNG::npr-28 3'UTR	Forward primer for cloning the *Pnpr-28* sequence	IDT	TTGCATGCCTGAGCTAATGTCCTATTCGCCCC
	Reverse primer for cloning the *Pnpr-28* sequence	IDT	TTGGAGACCATAGTAGAATAACTGACAAGTCGTGTAAAGCAAA
	Forward primer for cloning the mNG sequence	IDT	GTTATTCTACTATGGTCTCCAAGGGAGAGGAG
	Reverse primer for cloning the mNG sequence	IDT	AGTATTGTTATCTACTTGTAGAGCTCGTCCATTCCC
	Forward primer for cloning the *npr-28 3'UTR* sequence	IDT	TCTACAAGTAGATAACAATACTTTGAATATGTACCTTTTTTTCAACTTGTATCATT

bioRxiv preprint doi: https://doi.org/10.1101/2025.10.03.680215; this version posted October 4, 2025. The copyright holder for this preprint (which was not certified by peer review) is the author/funder, who has granted bioRxiv a license to display the preprint in perpetuity. It is made available under aCC-BY 4.0 International license.

	Reverse primer for cloning the *npr-28 3'UTR* sequence	IDT	TGAATTCGAGCTATTTCGTAATATTTATTTGTTTATCTTTTTATTTACTTATTTCACTCA
	Forward primer for cloning the pUC19 sequence	IDT	ATTACGAAATAGCTCGAATTCACTGGCCG
	Reverse primer for cloning the pUC19 sequence	IDT	GGACATTAGCTCAGGCATGCAAGCTTGGCGT
